# New Instrument for Operating on Hemorrhoids

**Published:** 1850-05

**Authors:** 


### SELECTIONS

FROM

### AMERICAN AND FOREIGN JOURNALS.

---

*New Instrument for Operating on Hemorrhoids.* By M. Amussat.—M. Amussat has invented a new instrument for the eradication of these troublesome companions. It is a small forceps, somewhat like a dissecting forceps.—The points, or rather the branches, for one-third of their length, are split, and receive two small hollow cylinders, which are charged with Vienna caustic at the moment of operation. The branches of the forceps are brought together by a screw, and the base of the pile attached by constriction and cauterization conjointly. When the instrument has acted for some time, a jet of cold acidulated water is directed on the part, to remove the superfluous caustic and allay the sensation of burning. M. Amussat has already applied his instrument in practice with complete success.—*Medical Times, July* 14, 1849, *p.* 28.

